# Interplays between auxin and GA signaling coordinate early fruit development

**DOI:** 10.1093/hr/uhab078

**Published:** 2022-01-19

**Authors:** Hai He, Chizuko Yamamuro

**Affiliations:** FAFU-UCR Joint Center for Horticultural Biology and Metabolomics, Haixia Institute of Science and Technology, Fujian Agriculture and Forestry University, Fuzhou 350002, Fujian, China

## Abstract

Phytohormones and their interactions are critical for fruit development and are key topics in horticulture research. Auxin, together with gibberellic acid (GA), promotes cell division and expansion, thereby regulating fruit development and enlargement after fertilization. Auxin- and GA-related mutants show parthenocarpy (fruit formation without fertilization of the ovule) in many plant species, indicating that these hormones and possibly their interactions play a key role in the regulation of fruit initiation and development. Recent studies have shown clear molecular and genetic evidence that ARF/IAA and DELLA proteins interact with one another and regulate both auxin and GA signaling pathways in response to auxin and GA during fruit growth in horticultural plants such as tomato (the most studied fleshy fruit) and strawberry (the model for Rosaceae). These recent findings provide new insights into the mechanisms by which the plant hormones auxin and GA regulate fruit development.

## Introduction

The plant hormone auxin, acting together with other plant hormones, is a key regulator of various developmental processes in plants. Hormonal control of cell division and expansion is especially important for early fruit development in horticultural plants. In 1788, Joseph Gaertner defined a fruit as a seed-containing structure derived from a mature ovary [1]. Fruits are often accompanied by accessory fruits, which contain other parts of the floral tissue [2, 3]. During flower development, the floral tissue (typically the gynoecium) that will develop into a fruit, is only a small portion of the floral organ. After flower opening, fertilization triggers the enlargement of this tissue, first through the action of auxin [4, 5]. The auxin signal can also promote the biosynthesis of other plant hormones such as gibberellin (GA), and the interaction of auxin with other hormones then coordinates fruit growth and development [6–9]. Auxin and GA promote cell division and expansion during plant development in many plant species [6–9]. Because of the diversity in the structure of floral organs or morphological features, fruits in different horticultural crops are not anatomically identical organs. However, it is interesting that the growth of these anatomically different fruit tissues is controlled by the same plant hormones auxin and GA and that only fruit tissues in floral organs have strong competence for enlargement in response to these plant hormones. Therefore, plant hormones such as auxin and GA are major regulators in controlling the enlargement of fruits of different plant species. Where (in which tissue) does auxin come from to promote fruit growth? Are the key players in the signaling cascades of these plant hormones conserved in anatomically different fruit? How are the functions of auxin and GA coordinated during early fruit development? In this review, we discuss how auxin and GA act coordinately to initiate fruit development and to promote fruit growth, focusing on the model plant *Arabidopsis thaliana* and two horticultural plants, tomato (*Solanum lycopersicum*) and strawberry (*Fragaria vesca*), as excellent examples of molecular genetic studies on fruit development. The role of GA in parthenocarpic fruit development is especially important in grape production, and we discuss the potential mechanism revealed by expression analysis in grape.

**Figure 1:**
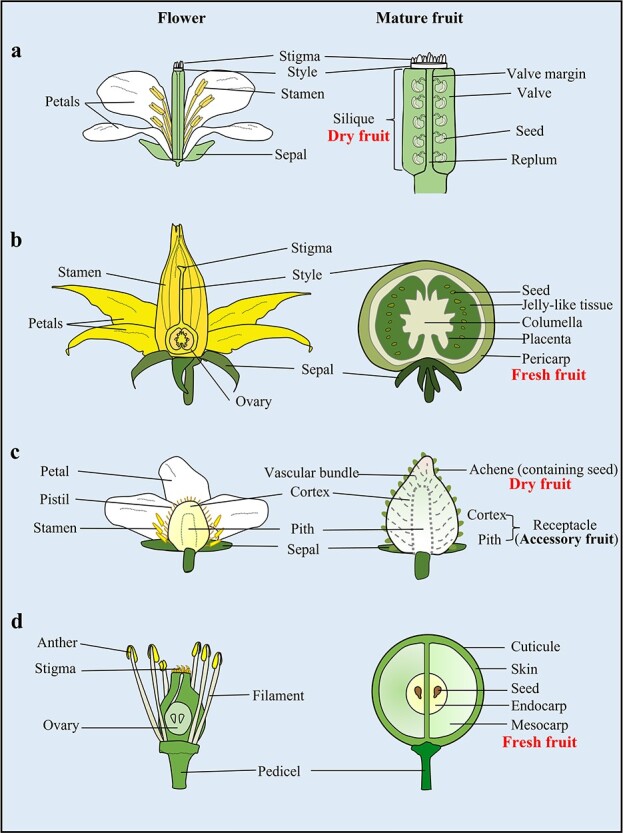
Schematic representation of longitudinal sections of flowers and fruits. **a** The Arabidopsis fruit is a silique formed from fused carpels. After fruit maturation, the valve margins differentiate into a dehiscence zone for fruit opening [13, 14]. **b** The tomato fruit is a fleshy fruit, a syncarpous gynoecium of several fused carpels with many seeds embedded in a fleshy mass [14–16]. **c** Strawberry has many individual pistils in a single flower, and the fleshy part of the strawberry is the receptacle (accessory fruit, which is the tip of the stem). The true fruit of the strawberry is a dry indehiscent fruit, the achene (the true fruit, which contains the seed) on the surface of the receptacle [17]. **d** The grape berry is a fleshy fruit, a syncarpous gynoecium of fused carpels. The ovary wall becomes the pericarp, which is composed of the skin, mesocarp, and endocarp [18].

## The anatomy of fruit and the functions of auxin and GA in fruit initiation and growth

The fruits of the angiosperms are derived from the gynoecium after fertilization of the ovule in the carpel [10–12] (Fig. 1). Fruits can be classified into dry fruit and fleshy fruit. Arabidopsis fruit is an example of a dry dehiscent fruit from which seeds are released when ripe. By contrast, tomato and grape are fleshy fruits. Seeds of tomato and grape are embedded in jelly-like tissue and endocarp, respectively [13–15]. Strawberry fruit is composed of a dry indehiscent fruit (the true fruit called the achene) and a receptacle (a large portion of the edible part) located at the base of the flower (the stem tip) [17]. It is well known that auxin from the achene promotes receptacle fruit growth after fertilization in strawberry. However, fruit development and enlargement can occur independent of fertilization and embryogenesis in a process known as parthenocarpy. Parthenocarpy is a valuable and important trait, especially in horticulture, because edible but seedless fruits attract consumers, and its underlying mechanisms have thus attracted a great deal of attention. In general, auxin and GA produced in developing seeds are important for early fruit growth [19–24]. It has also been shown that auxin and/or GA treatment promotes the formation of seedless fruits (parthenocarpic fruits) in many plant species [25–30]. Similarly, the defect in receptacle enlargement caused by the removal of achenes can be rescued by the application of auxin in strawberry [19]. How are these anatomically different fruit tissues controlled by the plant hormones auxin and GA during fruit development? Part of this question can be answered with molecular and genetic evidence from recent research on fruit development in different plant species, including Arabidopsis and the horticultural plants tomato, strawberry, and grape.

## Lessons from molecular mechanisms of auxin and GA signaling in model plant species

**Figure 2:**
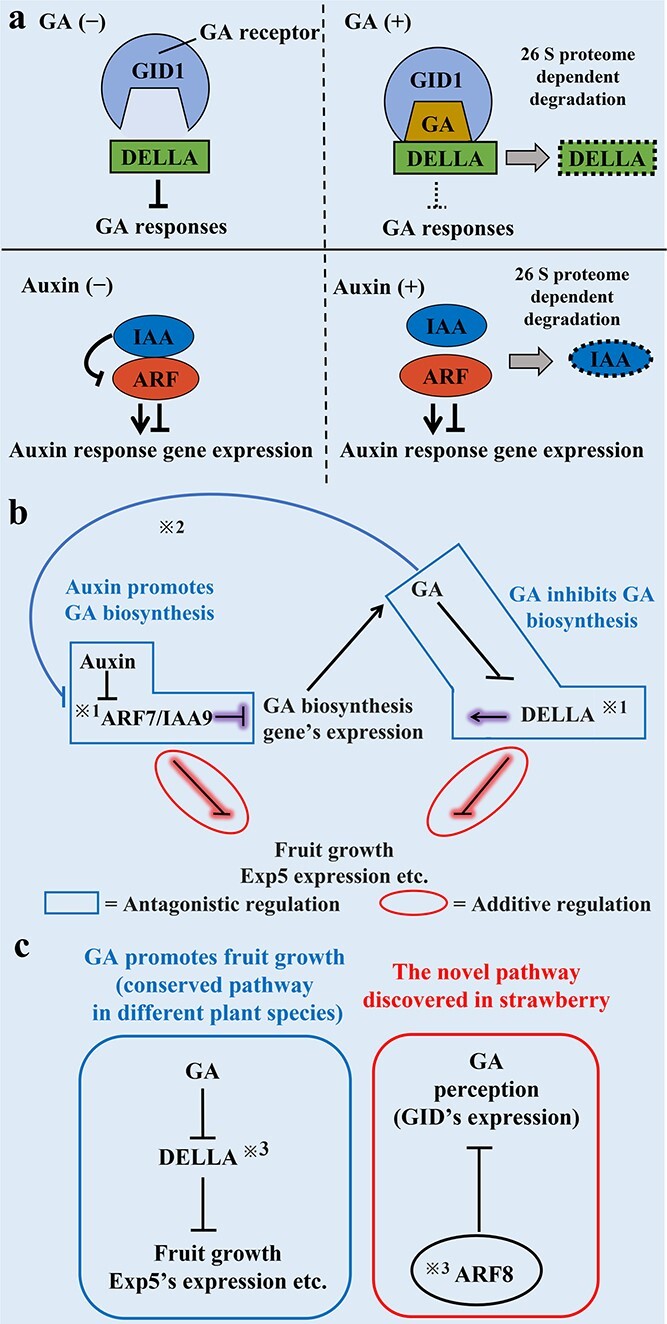
A model of DELLA and ARF/IAA function. **a** DELLA protein negatively regulates GA responses. In the presence of GA, the formation of a GA-GID1-DELLA complex leads to degradation of the DELLA protein by the 26S proteasome pathway, which promotes the expression of target genes (top panel). In the presence of auxin, the formation of an ARF-IAA complex leads to degradation of the IAA protein by the 26S proteasome pathway, which regulates the expression of auxin response genes (bottom panel). **b** The regulation of fruit growth by DELLA and the SlARF7/SlIAA9 pathway during tomato fruit development. DELLA and SlARF7/SlIAA9 regulate the GA biosynthesis pathway antagonistically. The additive regulation of fruit growth–related genes by DELLA and SlARF7/SlIAA9. 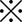
1, Protein–protein interaction between DELLA and SlARF7/SlIAA9. 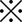
2, GA treatment significantly reduced the enrichment of SlARF7 at the *EXP5* locus. **c** DELLA and FveARF8 function in strawberry accessory fruit development. FveARF8 inhibits the expression of *FveGID* in strawberry accessory fruit development. 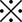
3, Protein–protein interactions between FveARF8 and DELLA.

The molecular mechanisms of the auxin and GA signaling pathways have been well studied, especially in model plant species such as Arabidopsis. Auxin and GA are perceived by receptor proteins to regulate their downstream pathways. The regulation of gene expression by auxin is directly controlled by ARF (auxin response factor) transcription factor proteins. In the absence of auxin, ARF function is restricted by the direct binding of the repressor protein IAA (INDOLE-3-ACETIC ACID INDUCIBLE) through its domains III and IV. Once auxin is perceived by the nuclear auxin receptor F-box protein TIR1/AFB (TRANSPORT INHIBITOR RESPONSE 1/AUXIN-SIGNALING F-BOX), ubiquitination-dependent IAA protein degradation occurs, leading to the activation of ARF proteins (Fig. 2a) [31–34]. The ARF proteins form large gene families in higher plants and can be divided into three subgroups, types I, II, and III. ARFs from different subgroups act as activators or repressors of the transcription of their target genes [35, 36]. Interestingly, the Arabidopsis recessive *arf8* mutant (ARF8 is an activator ARF) exhibited fruit growth independent of fertilization signals, indicating that the activator ARF8 negatively regulates fruit initiation and development in Arabidopsis [37, 38].

The molecular basis of the GA signaling pathway is also well characterized in model plant species. GA signaling is negatively regulated by the master repressor DELLA proteins, which interrupt transcription factors such as PIF4, encoded by *PHYTOCHROME INTERACTING FACTOR 4*, by direct binding [39–43]. The proteasome-dependent degradation of DELLA protein is enhanced by the binding of GIBBERELLIN INSENSITIVE DWARF1 (GID1) and DELLA protein in a GA-dependent manner (Fig. 2a) [44–46]. It is known that five Arabidopsis DELLA proteins have functional redundancy to suppress GA signaling. Analysis of a quadruple DELLA mutant showed that *RGA* (*REPRESSOR OF GA*), *GAI* (*GA INSENSITIVE)*, *RGL1* (*RGA-LIKE 1*), and *RGL2* are important for the suppression of GA signaling in fruit initiation and growth because of the strong parthenocarpic phenotype of the mutant [7, 47]. Interestingly, significant fruit growth can be further promoted by GA treatment in the quintuple DELLA mutant (lacking all five DELLA proteins) in specific cell layers [47]. The DELLA-independent GA response requires the GID receptor and the 26S proteasome [47]. This result revealed the role of the DELLA-independent GA response in fruit development. The DELLA-independent pathway depends on the basic helix–loop–helix transcription factor SPT (SPATULA) [47]. The biological role of the DELLA-independent pathway in fruit growth has not been shown. However, the authors proposed the possibility that the DELLA-independent pathway provides additional opportunities for fine-tuning fruit growth [47]. Both auxin and GA promote fruit growth. How do these plant hormones coordinately regulate fruit growth and development? The basic auxin and GA signaling pathways and their importance in fruit development have been studied in Arabidopsis, but some answers to this question, especially the interplay between auxin and GA, have come from studies of fruit development in horticultural plants, as described in the following sections.

## Molecular and genetic studies of auxin and GA actions in tomato fruit development

Clear differences in the traits of auxin- and GA-treated fruit have been observed since early studies, although both auxin and GA promote tomato fruit development. For example, jelly-like tissue development was almost absent in GA-induced parthenocarpic fruit, whereas auxin treatment produced pseudoembryos and increased the number of vascular bundles in auxin-induced parthenocarpic fruit [48], indicating different roles of these plant hormones in fruit development. How do these two hormones coordinate tomato fruit growth? It has long been known that auxin can promote GA biosynthesis in many plant species. Indeed, the expression of GA biosynthesis genes (*GA20ox* and/or *GA3ox)* is promoted by auxin treatment in tomato fruit [49–52], suggesting that some aspects of auxin function occur through GA. Interestingly, initiation of fruit growth by auxin treatment was not observed in the severely GA-deficient *gib1* mutant background in tomato. This result indicates that the function of auxin in fruit growth is GA dependent in tomato [53]. Importantly, the same phenomenon of GA-dependent auxin function in parthenocarpy was also observed in Arabidopsis. However, GA-dependent RGA1 (DELLA) protein degradation required auxin function in Arabidopsis [54]. If this auxin-mediated DELLA protein degradation is conserved in tomato fruit tissue, there must be a hierarchical interplay between auxin and GA to coordinate fruit development. The interplay may provide a mechanism to enable flexible fruit growth in response to the environment. However, the detailed mechanisms underlying this interplay remain unclear.

To understand the complex relationship between auxin and GA in fruit development, the molecular study of the signaling cascades of these plant hormones is necessary. The molecular mechanism of auxin signaling during parthenocarpic fruit development has been extensively studied in tomato as a model for horticultural plants. Early studies revealed that the knockout of *SlIAA9* and the knockdown of the activator ARF *SlARF7* (*SlRNA RNAi*: *ARF6*, *7*, and *8*; other activator *ARF* genes were also downregulated in the line) cause parthenocarpic fruits, suggesting that *SlIAA9* and *SlARF7* act as suppressors of fruit growth in non-pollinated fruit [53, 55, 56]. The negative regulation of fruit development by these suppressors seemed to be released after the fertilization of ovules, resulting in the promotion of fruit growth. Consistently, the downregulation of *SlARF7* expression was observed 48 h after pollination [6, 57, 58]. Both auxin and GA induce parthenocarpic fruit, suggesting the importance of these hormones in fruit development [53]. It has been discussed that SIARF7 may function in the interaction between auxin and GA signaling cascades during fruit development [6]. On the other hand, other studies suggest that DELLA proteins are also involved in the crosstalk between auxin and GA during fruit development. Knockout or downregulation of a single *DELLA* gene, *PROCERA*, leads to parthenocarpy, as observed in multiple knockout mutants of DELLA genes in Arabidopsis [7, 9, 59, 60]. Notably, an interaction between RGA (a DELLA protein) and the ARF proteins ARF6, 7, and 8 was observed in Arabidopsis [61]. These results strongly suggested that the complex of ARF and DELLA proteins acts as a crosstalk point between auxin and GA signaling. These studies raised an important question. If ARF and DELLA are in the same complex, how can the specific roles of each plant hormone in fruit initiation and growth be achieved? An excellent publication from Taiping Sun’s group in tomato may help to answer this question. In addition to direct interaction between SlARF7 and PROCERA (DELLA), the authors showed that SlARF7 and PROCERA directly regulate the same set of genes antagonistically or additively. Auxin promotes, but GA inhibits, the expression of GA biosynthesis genes through SlARF7 and PROCERA, respectively. Both SlARF7 and PROCERA can directly interact with the promoters of GA biosynthesis genes. Dual luciferase assays in *N. benthamiana* showed that PROCERA can clearly upregulate GA biosynthesis gene expression, but co-expression of SlARF7 and SlIAA9 significantly suppressed GA biosynthesis gene expression, suggesting that GA and auxin directly regulate GA biosynthesis gene expression through these factors [53]. Interestingly, the authors showed that SlARF7 negatively controls gene expression only when SlIAA9 is co-expressed with SlARF7. Because the negative regulation by SlARF7/SlIAA9 can be removed by auxin signaling through IAA degradation (auxin negatively regulates SlARF7/SlIAA9), auxin can thus promote the expression of GA biosynthesis genes (Fig. 2b). This result offers one explanation for why the activator SlARF7 can suppress gene expression. Furthermore, co-expression of PROCERA with SlARF7 and SlIAA9 (triple expression) clearly restored the suppression of GA biosynthesis genes by SlARF7/SlIAA9. These experiments explain the relationship between SlARF7/SlIAA9 and PROCERA in the antagonistic regulation of GA biosynthesis genes (Fig. 2b). On the other hand, SlARF7/SlIAA9 and PROCERA additively regulate the expression of other genes, such as *EXPANSIN5* (*EXP5*)*,* which is important for fruit growth [41] (Fig. 2b). PROCERA and SlARF7/SlIAA9 negatively regulate *EXP5* gene expression by direct binding to its promoter [53]. GA promotes *EXP5* expression and fruit growth. GA treatment releases the suppression signal of PROCERA for *EXP5* gene expression by promoting the degradation of PROCERA [53]. Furthermore, the authors found that GA treatment slightly reduced enrichment of SlARF7 at the *EXP5* locus, suggesting that PROCERA may enhance the direct binding of another negative regulator, SlARF7, to the *EXP5* locus. Together, these results provided strong evidence that the interaction between PROCERA, SlARF7, and SlIAA9 coordinates auxin and GA functions in fruit initiation [41]. How do SlARF7/SlIAA9 and PROCERA regulate target genes antagonistically or additively? Further analysis will be necessary to answer this question. Initially, the direct interaction between activator ARFs and DELLA was shown in Arabidopsis, but the biological importance of this interaction was further confirmed by the study of tomato. Interestingly, it is also known that cytokinin (CK) can induce parthenocarpy in tomato. The CK-induced parthenocarpy is dependent on auxin and GA biosynthesis [62]. This result also suggested the importance of auxin and GA function in early fruit development.

Studies in horticultural plants have the potential to reveal novel molecular mechanisms that can explain the interplay between plant hormones in fruit development. In addition, several reports that further support ARF and IAA function in tomato fruit development are summarized in Table 1.

**Table 1:** *ARF* and *Aux/IAA* genes in tomato and strawberry fruit development. *ARF* genes are classified as activators or repressors based on their Arabidopsis homologs.

**Activator or repressor**	**Gene name**	**Biological function (or process)**	**Phenotype**	**References**
Repressor	*SlARF2*	Regulator of fruit ripening	Delayed ripening phenotype	[63]
Repressor	*SlARF3*	Regulator of epidermal cell development	Decreased density of trichomes and pavement cells	[64]
Repressor	*SlARF4*	Regulator of chlorophyll and starch accumulation in fruit	Dark green and blotchy ripening fruit	[65]
Activator	*SlARF5*	Cell division and expansion during early fruit development	**Parthenocarpic** fruit	[66]
Activator	*SlARF6*	Photosynthesis, sugar accumulation, and fruit development	Delay of fruit ripening	[67]
Activator	*SlARF7*	Auxin and GA signaling response pathways in fruit development	**Parthenocarpic** fruit	[6]
Activator	*SlARF8*	Auxin and GA signaling response pathways in fruit development	**Parthenocarpic** fruit	[68]
Repressor	*SlARF9*	Auxin-responsive, cell division activity	Small fruit size	[69]
Repressor	*SlARF10*	Chlorophyll and sugar accumulation	Dark green fruit color	[70]
Repressor	*SlIAA3*	Auxin and ethylene signaling pathways	Reduced apical dominance, lower auxin sensitivity	[71]
Repressor	*SlIAA9*	Mediator of auxin signaling in fruit set and leaf morphogenesis	**Parthenocarpic** fruit	[55, 68]
-	*SlIAA27*	Ethylene and auxin signaling	Slightly increased plant size	[72]
-	*FveARF8*	Interplay between auxin and GA function in fruit development	Large fruit size	[73]

## Molecular and genetic studies of auxin and GA function in strawberry fruit development

As in tomato fruit development, auxin plays an important role in the initiation and growth of receptacle fruits in strawberry. It has long been known that auxin from the achenes promotes receptacle fruit growth after fertilization in strawberry [5]. Compared with studies in tomato, molecular studies of auxin functions in strawberry fruit development are still limited. However, auxin function and gene expression changes in response to auxin treatment in strawberry fruits have long been discussed in cultivated strawberry [74, 75]. Recently, the diploid strawberry *Fragaria vesca* has become an exciting model system for the molecular study of strawberry fruit development because of its small genome size, short life cycle, and other traits. Auxin levels and auxin signaling activity reported by *DR5p:GUS* expression were shown to be particularly high in the early stage of fruit development after fertilization [8, 76]. In agreement with the earlier observation that achenes supply plant hormones to promote fruit growth, auxin and GA biosynthesis genes were shown to be expressed mainly in an achene tissue known as ghost [26], whereas the expression of auxin- and GA-induced genes and signaling genes such as *ARFs*, *GIDs*, and DELLAs (*GAI*, *RGA*) was localized mainly in receptacle fruits [26]. This result is consistent with the general idea that plant hormones from the achenes play a role in the strawberry receptacle.

Given the crosstalk of auxin with many different plant hormones [77], it is not surprising that auxin treatment promotes the expression of the GA biosynthesis genes *FveGA20OX* and *FveGA3OX* in the early stage of strawberry fruit growth [8]. Both auxin and GA promote fruit growth, but auxin and GA have clearly different effects on fruit shape formation and strawberry fruit development [8, 26]. Auxin treatments produce round fruit, whereas GA-treated fruits exhibit a longer shape [8]. These results suggest that auxin and GA have different roles in strawberry fruit development. As discussed above (in the sections on tomato and Arabidopsis), auxin and GA signaling pathways are connected by a complex regulatory mechanism. Detailed molecular study of the interaction between auxin and GA will be important for understanding the function of each plant hormone in fruit development.

As mentioned above, strawberry (receptacle) and tomato fruit (or Arabidopsis) are anatomically different tissues, but fruit growth in both species is promoted by exogenous application of auxin and GA. An interesting question is whether the signaling cascades for auxin and GA and the mechanism for their interplay observed in other plant species are also conserved in strawberry receptacle fruit development. Recently, an excellent publication provided one clue to answer the question with strong genetic evidence. *FveRGA1* was previously reported to be a negative regulator of runner development [78]. Zhongchi Liu’s group further showed a role for *FveRGA1* in fruit development. The knockout of *FveRGA1* produced a parthenocarpic phenotype, suggesting that *FveRGA1* is a key regulator of fruit initiation in strawberry [73]. The conserved interaction of FveRGA1 and FveARF8 was also shown in yeast and tobacco cells [73]. Fruits were wider in a knockout mutant of *FveARF8*, suggesting the negative regulation of fruit growth through FveARF8 (the Arabidopsis homolog was originally characterized as an activator ARF) in early fruit growth. Interestingly, the authors found that FveARF8 directly binds to the promoter of *GID1c* (GA receptor gene *FveGID1c*) and negatively controls its expression [73]. The authors suggested a novel mechanism that coordinates the interaction of the auxin and GA signaling cascades in strawberry fruit development (Fig. 2c). The interaction between strawberry FveARF8 and FveIAA4 was also confirmed [73]. An *FveARF8* knockout mutant did not show parthenocarpy in strawberry, and other activator ARFs such as FveARF5, 6, and 7 may have functional redundancy. Interestingly, the activator ARFs act as negative regulators of fruit growth in different plant species, and ARFs/IAA and DELLA proteins may therefore negatively control fruit growth and development in strawberry, as in other species. However, the precise mechanisms by which these auxin and GA signaling components regulate fruit development and by which auxin and GA coordinate their specific roles in fruit development remain to be fully elucidated.

## Plant hormone function in early grape berry development

GA promotes parthenocarpy and is widely used to produce seedless fruits in many different grape cultivars [27–29, 79–81]. The expression patterns of GA biosynthesis- and signaling-related genes in the grape berry have been well documented [82–84]. Wang et al. proposed that GA biosynthesis exceeds GA inactivation during the early stage of development [84]. Consistent with this observation, a dramatic increase in GA contents was observed during the green-berry stage. Auxin and CK also promote parthenocarpy in grape [85, 86]. In addition to GA, auxin and CK contents are also relatively high in the early stage of grape berry development [87]. Wang et al. proposed a potential crosstalk between GA and other plant hormones such as auxin and CK by precise transcriptomic profiling [84]. These results support the important roles of GA, auxin, and CK in early grape berry development. Furthermore, another transcriptome analysis also proposed that auxin plays an important role in regulating cell division and cell expansion in grape berry development [88]. Interestingly, the grape berry growth induced by auxin and CK treatment is also partially dependent on GA biosynthesis [86], suggesting the importance of the interplay among these plant hormones. Are the molecular mechanisms for the functions of ARF/IAA and DELLA observed in tomato and/or strawberry also conserved in grape berry development? GA treatment negatively regulates the expression of *VvIAA9* and *VvARF7* at the pre-bloom stage in grape [89]. Furthermore, Wang et al. performed spatiotemporal expression analysis of *VvmiR159*, *VvGAMYB* (the direct target of *VvmiR159*), and DELLA genes in early fruit development. The authors proposed an attractive model in which GA-DELLA-*VvmiR159c*-*VvGAMYB* is the key signaling regulatory module in grape based on the expression patterns of these genes in early fruit development [90]. Further genetic analysis will be needed to show clear molecular mechanisms of plant hormone function and plant hormone interactions in grape berry development.

## Conclusion and perspective

The early stage of fruit development is especially important for fruit production. In this review, we mainly discuss crosstalk between auxin and GA function in early fruit development of tomato and strawberry. Recent studies have provided molecular evidence for the interplay between auxin and GA, supporting basic knowledge gained in the earlier years of horticultural research. The many important players in fruit development seem to be conserved in different plant species. DELLA proteins, which act as one of the major regulators of fruit development, play a role as key proteins in signaling cascades that regulate many important biological processes, including plant hormone interactions. In this context, we expect that further study of the ARF/IAA and DELLA protein complex will be especially important for further understanding of plant hormone functions in fruit development. In addition to GA and auxin, DELLA proteins are important integrators of other plant hormones such as ethylene, abscisic acid, brassinosteroid, and jasmonic acid [91–93]. DELLAs may be key proteins that coordinate these plant hormone functions in fruit development. Tomato has only one DELLA protein, whereas strawberry has five DELLA proteins, although only one has a complete DELLA domain [26, 78]. Because there is little or no functional redundancy for DELLA proteins in tomato and strawberry, in-depth studies of the molecular functions of DELLA in fruit development in these species may provide important paradigms for understanding the mechanisms underlying fruit development in other horticultural crops. Taking advantage of this feature in tomato and strawberry, we anticipate that the investigations of hormonal control of fruit development in these species will be prolific in the years to come. Moreover, mechanistic studies of how fruit tissue (anatomically different tissues in different plant species) obtains the competence to respond to hormones that promote fruit tissue enlargement will generate fundamental insights into the common mechanisms that underlie fruit development in plants. Genome tools and basic knowledge obtained in model horticultural plants have no doubt impacted our understanding of fruit development at the molecular level. Furthermore, research on horticultural crops, such as the molecular and genetic studies of fruit development outlined here, has the potential not only to improve the production of horticultural plants but also to build greater and deeper understanding of basic science because of the diversity of horticultural plant species. The study of the interplay between auxin and GA in fruit development is a good example. The excellent publications discussed here are initial accomplishments that open the way for future advances in plant science.

## References

[1] Gaertner J . De Fructibus et Seminibus Plantarum. Stuttgart: typis Academiae Carolinae; 1788.

[2] Esau K . Plant Anatomy. N.Y: John Wiley and Sons Press; 1967.

[3] Weberling F . Morphology of Flowers and Inflorescences. United Kingdom: Cambridge Univ. Press; 1989.

[4] Gillaspy G , Ben-David H, Gruissem W. Fruits: a developmental perspective. Plant Cell. 1993;5:1439–51. 12271039 10.1105/tpc.5.10.1439 PMC160374

[5] Nitsch JP . Growth and morphogenesis of the strawberry as related to auxin. Am J Bot. 1950;37:211–5.

[6] de Jong M , Wolters-Arts M, García-Martínez J et al. The *Solanum lycopersicum* AUXIN RESPONSE FACTOR 7 (SlARF7) mediates cross-talk between auxin and gibberellin signaling during tomato fruit set and development. J Exp Bot. 2011;62:617–26. 20937732 10.1093/jxb/erq293 PMC3003806

[7] Dorcey E , Urbez C, Carbonell J et al. Fertilization-dependent auxin response in ovules triggers fruit development through the modulation of gibberellin metabolism in Arabidopsis. Plant J. 2009;58:318–32. 19207215 10.1111/j.1365-313X.2008.03781.x

[8] Liao X , Li M, Liu B et al. Interlinked regulatory loops of ABA catabolism and biosynthesis coordinate fruit growth and ripening in woodland strawberry. Proc Natl Acad Sci U S A. 2018;115:201812575. 10.1073/pnas.1812575115 PMC6298082 30455308

[9] Ozga JA , Reinecke DM, Ayele BT et al. Developmental and hormonal regulation of gibberellin biosynthesis and catabolism in pea fruit. Plant Physiol. 2009;150:448–62. 19297588 10.1104/pp.108.132027 PMC2675736

[10] Knoll F . Über den Begriff «Frucht». Der Biologe. 1939;8:154–60.

[11] Roth I . Fruits of angiosperms. Berlin: Gebrüder Borntraeger Press; 1977.

[12] Cutler DF , Botha CEJ, Stevenson DW. Plant anatomy: An applied approach. United Kingdom: Blackwell press; 2008.

[13] Seymour GB , Østergaard L, Chapman NH et al. Fruit development and ripening. Annu Rev Plant Biol. 2013;64:219–41. 23394500 10.1146/annurev-arplant-050312-120057

[14] Gasser SE , Robinson-Beers K. Pistil development. Plant Cell. 1993;5:1231–9. 12271024 10.1105/tpc.5.10.1231 PMC160356

[15] Pabon-Mora N , Litt A. Comparative anatomical and developmental analysis of dry and fleshy fruits of Solanaceae. Am J Bot. 2011;98:1415–36. 21875970 10.3732/ajb.1100097

[16] Ho LC , Hewitt JD. Fruit development. In: Atherton, JG and Rudich J eds. The Tomato Crop. NY: Chapman and Hall; 1986;201–39.

[17] Hollender CA , Geretz AC, Slovin JP et al. Flower and early fruit development in a diploid strawberry. *Fragaria vesca* Planta. 2011;235:1123–39. 22198460 10.1007/s00425-011-1562-1

[18] Conde C , Silva PF, Dias ACP et al. Biochemical changes throughout grape berry development and fruit and wine quality. FoodReview. 2007;1:1–22.

[19] Archbold DD , Dennis FG. Strawberry receptacle growth and endogenous IAA content as affected by growth regulator application and achene removal. J Am Soc Hortic Sci. 1985;110:816–20.

[20] Picken AJF . A review of pollination and fruit set in the tomato (Lycopersicon esculentum mill.). Journal of Horticultural Science. 1984;59:1–13.

[21] Gustafson FG . Inducement of fruit development by growth-promoting chemicals. Proc Natl Acad Sci U S A. 1936;22:628–36. 16577739 10.1073/pnas.22.11.628 PMC1076829

[22] Gustafson FG . The natural cause of parthenocarpy. Am J Bot. 1939;26:135–8.

[23] Nitsch JP . Growth and morphogenesis of the strawberry as related to auxin. Am J Bot. 1950;37:211–5.

[24] Nitsch JP . Plant hormones in the development of fruit. Q Rev Biol. 1952;27:33–57. 14930223 10.1086/398643

[25] Gorguet B , van Heusden AW, Lindhout P. Parthenocarpic fruit development in tomato. Plant Biol. 2005;7:131–9. 15822008 10.1055/s-2005-837494

[26] Kang C , Darwish O, Geretz A et al. Genome-scale transcriptomic insights into early-stage fruit development in woodland strawberry *Fragaria vesca*. Plant Cell. 2013;25:1960–78. 23898027 10.1105/tpc.113.111732 PMC3723606

[27] Cheng C , Jiao C, Singer et al. Gibberellin-induced changes in the transcriptome of grapevine (*Vitis labrusca* × *V. vinifera*) cv. Kyoho flowers. BMC Genomics. 2015;16:128. 25888129 10.1186/s12864-015-1324-8 PMC4348105

[28] Gouthu S , Deluc LG. Timing of ripening initiation in grape berries and its relationship to seed content and pericarp auxin levels. BMC Plant Biol. 2015;15:46. 25848949 10.1186/s12870-015-0440-6 PMC4340107

[29] Upadhyay A , Jogaih S, Kadoo N et al. Expression of stable reference genes and *SPINDLY* gene in response to gibberellic acid application at different stages of grapevine development. Bio plant. 2015;59:436–44.

[30] McAtee P , Karim S, Schaffer R et al. Dynamic interplay between phytohormones is required for fruit development, maturation, and ripening. Front Plant Sci. 2013;4:79. 23616786 10.3389/fpls.2013.00079 PMC3628358

[31] Dharmasiri N , Dharmasiri S, Estelle M. The F-box protein TIR1 is an auxin receptor. Nature. 2005;435:441–5. 15917797 10.1038/nature03543

[32] Kepinski S , Leyser O. The Arabidopsis F-box protein TIR1 is an auxin receptor. Nature. 2005;435:446–51. 15917798 10.1038/nature03542

[33] Mockaitis K , Estelle M. Auxin receptors and plant development: a new signaling paradigm. Annu Rev Cell Dev Biol. 2008;24:55–80. 18631113 10.1146/annurev.cellbio.23.090506.123214

[34] Salehin M , Bagchi R, Estelle M. SCFTIR1/AFB-based auxin perception: mechanism and role in plant growth and development. Plant Cell. 2015;27:9–19. 25604443 10.1105/tpc.114.133744 PMC4330579

[35] Ulmasov T , Hagen G, Guilfoyle TJ. Dimerization and DNA binding of auxin response factors. Plant J. 1999a;19:309–19. 10476078 10.1046/j.1365-313x.1999.00538.x

[36] Ulmasov T , Hagen G, Guilfoyle TJ. Activation and repression of transcription by auxin-response factors. Proc Natl Acad Sci U S A. 1999b;96:5844–9. 10318972 10.1073/pnas.96.10.5844 PMC21948

[37] Vivian-Smith A , Adam. The molecular basis for the initiation of fruit development and parthenocarpy. Theses. 2001.

[38] Goetz M , Vivian-Smith A, Johnson SD et al. AUXIN RESPONSE FACTOR8 is a negative regulator of fruit initiation in Arabidopsis. Plant Cell. 2006;18:1873–86. 16829592 10.1105/tpc.105.037192 PMC1533983

[39] Davière JM , Achard P. A pivotal role of DELLAs in regulating multiple hormone signals. Mol Plant. 2016;9:10–20. 26415696 10.1016/j.molp.2015.09.011

[40] de Lucas M , Daviere JM, Rodriguez-Falcon M et al. A molecular framework for light and gibberellin control of cell elongation. Nature. 2008;451:480–4. 18216857 10.1038/nature06520

[41] Peng J , Carol P, Richards DE et al. The Arabidopsis *GAI* gene defines a signaling pathway that negatively regulates gibberellin responses. Genes Dev. 1997;11:3194–205. 9389651 10.1101/gad.11.23.3194 PMC316750

[42] Silverstone AL , Ciampaglio CN, Sun TP. The Arabidopsis RGA gene encodes a transcriptional regulator repressing the gibberellin signal transduction pathway. Plant Cell. 1998;10:155–69. 9490740 10.1105/tpc.10.2.155 PMC143987

[43] Sun TP . Gibberellin-GID1-DELLA: a pivotal regulatory module for plant growth and development. Plant Physiol. 2010;154:567–70. 20921186 10.1104/pp.110.161554 PMC2949019

[44] Mcginnis KM , Thomas SG, Soule JD et al. The Arabidopsis *SLEEPY1* gene encodes a putative F-box subunit of an SCF E3 ubiquitin ligase. Plant Cell. 2003;15:1120–30. 12724538 10.1105/tpc.010827 PMC153720

[45] Murase K , Hirano Y, Sun TP et al. Gibberellin-induced DELLA recognition by the gibberellin receptor GID1. Nature. 2008;456:459–63. 19037309 10.1038/nature07519

[46] Ueguchi-Tanaka M , Ashikari M, Nakajima M et al. GIBBERELLIN INSENSITIVE DWARF1 encodes a soluble receptor for gibberellin. Nature. 2005;437:693–8. 16193045 10.1038/nature04028

[47] Fuentes S , Ljung K, Sorefan K et al. Fruit growth in Arabidopsis occurs via DELLA-dependent and DELLA-independent gibberellin responses. Plant Cell. 2012;24:3982–96. 23064323 10.1105/tpc.112.103192 PMC3517231

[48] Serrani JC , Fos M, Atarés A et al. Effect of gibberellin and auxin on parthenocarpic fruit growth induction in the cv micro-tom of tomato. J Plant Growth Regul. 2007;26:211–21.

[49] Frigerio M , Alabadi D, Perez-Gomez J et al. Transcriptional regulation of gibberellin metabolism genes by auxin signaling in Arabidopsis. Plant Physiol. 2006;142:553–63. 16905669 10.1104/pp.106.084871 PMC1586059

[50] Ross JJ , O’Neill DP, Smith JJ et al. Evidence that auxin promotes gibberellin A1 biosynthesis in pea. Plant J. 2000;21:547–52. 10758505 10.1046/j.1365-313x.2000.00702.x

[51] Wolbang CM , Chandler PM, Smith JJ et al. Auxin from the developing inflorescence is required for the biosynthesis of active gibberellins in barley stems. Plant Physiol. 2004;134:769–76. 14730077 10.1104/pp.103.030460 PMC344552

[52] Wolbang CM , Ross JJ. Auxin promotes gibberellin biosynthesis in decapitated tobacco plants. Planta. 2001;214:153–7. 11762165 10.1007/s004250100663

[53] Hu J , Alon I, Naomi O et al. The interaction between DELLA and ARF/IAA mediates crosstalk between gibberellin and auxin signaling to control fruit initiation in tomato. Plant Cell. 2018;30:1710–28. 30008445 10.1105/tpc.18.00363 PMC6139683

[54] Fu X , Harberd NP. Auxin promotes Arabidopsis root growth by modulating gibberellin response. Nature. 2003;421:740–3. 12610625 10.1038/nature01387

[55] Wang H , Jones B, Li Z et al. The tomato aux/IAA transcription factor IAA9 is involved in fruit development and leaf morphogenesis. Plant Cell. 2005;17:2676–92. 16126837 10.1105/tpc.105.033415 PMC1242265

[56] Zhang JH , Chen R, Xio J et al. A single-base deletion mutation in SlIAA9 gene causes tomato (*Solanum lycopersicum*) entire mutant. J Plant Res. 2007;120:671–8. 17955175 10.1007/s10265-007-0109-9

[57] de Jong M , Wolters-Arts M, Feron R et al. *Solanum lycopersicum* auxin response factor 7 (*Sl*ARF7) regulates auxin signaling during tomato fruit set and development. Plant J. 2009;57:160–70. 18778404 10.1111/j.1365-313X.2008.03671.x

[58] Vriezen WH , Feron R, Maretto F et al. Changes in tomato ovary transcriptome demonstrate complex hormonal regulation of fruit set. New Phytol. 2008;177:60–76. 18028300 10.1111/j.1469-8137.2007.02254.x

[59] Serrani JC , Ruiz-Rivero O, Fos M et al. Auxin-induced fruit-set in tomato is mediated in part by gibberellins. Plant J. 2008;56:922–34. 18702668 10.1111/j.1365-313X.2008.03654.x

[60] Carrera E , Ruiz-Rivero O, Peres LE et al. Characterization of the procera tomato mutant shows novel functions of the SIDELLA protein in the control of flower morphology, cell division and expansion, and the auxin-signaling pathway during fruit-set and development. Plant Physiol. 2012;160:1581–96. 22942390 10.1104/pp.112.204552 PMC3490602

[61] Oh E , Zhu JY, Bai MY et al. Cell elongation is regulated through a central circuit of interacting transcription factors in the Arabidopsis hypocotyl. elife. 2014;3:e03031. 10.7554/eLife.03031 PMC4075450 24867218

[62] Ding J , Chen B, Xia X et al. Cytokinin-induced parthenocarpic fruit development in tomato is partly dependent on enhanced gibberellin and auxin biosynthesis. PLoS One. 2013;8:e70080. 23922914 10.1371/journal.pone.0070080 PMC3726760

[63] Breitel DA , Chappell-Maor L, Meir S et al. AUXIN RESPONSE FACTOR 2 intersects hormonal signals in the regulation of tomato fruit ripening. PLoS Genet. 2016;12:e1005903. 26959229 10.1371/journal.pgen.1005903 PMC4784954

[64] Zhang X , Yan F, Tang Y et al. Auxin response gene *SlARF3* plays multiple roles in tomato development and is involved in the formation of epidermal cells and trichomes. Plant Cell Physiol. 2015;56:2110–24. 26412778 10.1093/pcp/pcv136

[65] Sagar M , Chervin C, Mila I et al. *SlARF4,* an auxin response factor involved in the control of sugar metabolism during tomato fruit development. Plant Physiol. 2013;161:1362–74. 23341361 10.1104/pp.113.213843 PMC3585602

[66] Liu S , Zhang Y, Feng Q et al. Tomato AUXIN RESPONSE FACTOR 5 regulates fruit set and development via the mediation of auxin and gibberellin signaling. Sci Rep. 2018;8:2971. 29445121 10.1038/s41598-018-21315-y PMC5813154

[67] Yuan Y , Xu X, Gong Z et al. Auxin response factor 6A regulates photosynthesis, sugar accumulation, and fruit development in tomato. Hortic Res. 2019;6:85. 31645946 10.1038/s41438-019-0167-x PMC6804849

[68] Goetz M , Hooper LC, Johnson SD et al. Expression of aberrant forms of AUXIN RESPONSE FACTOR8 stimulates parthenocarpy in Arabidopsis and tomato. Plant Physiol. 2007;145:351–66. 17766399 10.1104/pp.107.104174 PMC2048734

[69] de Jong M , Wolter-Arts M, Schimmel BCJ et al. *Solanum lycopersicum* AUXIN RESPONSE FACTOR 9 regulates cell division activity during early tomato fruit development. J Exp Bot. 2015;66:3405–16. 25883382 10.1093/jxb/erv152 PMC4449553

[70] Yuan Y , Mei L, Wu M et al. SlARF10, an auxin response factor, is involved in chlorophyll and sugar accumulation during tomato fruit development. J Exp Bot. 2018;69:5507–18. 30219898 10.1093/jxb/ery328 PMC6255703

[71] Chaabouni S , Jones B, Delalande C et al. Sl-IAA3, a tomato aux/IAA at the crossroads of auxin and ethylene signalling involved in differential growth. J Exp Bot. 2009;60:1349–62. 19213814 10.1093/jxb/erp009 PMC2657550

[72] Liu M , Chen Y, Chen Y et al. The tomato ethylene response factor Sl-ERF.B3 integrates ethylene and auxin signaling via direct regulation of *Sl-aux/IAA27*. New Phytol. 2018;219:631–40. 29701899 10.1111/nph.15165

[73] Zhou J , Sittmann J, Guo L et al. Gibberellin and auxin signaling genes RGA1 and ARF8 repress accessory fruit initiation in diploid strawberry. Plant Physiol. 2021;185:1059–75. 33793929 10.1093/plphys/kiaa087 PMC8133647

[74] Harpster MH , Brummell DA, Dunsmuir P. Expression analysis of a ripening-specific, auxin-repressed endo-1, 4-beta-glucanase gene in strawberry. Plant Physiol. 1998;118:1307–16. 9847104 10.1104/pp.118.4.1307 PMC34746

[75] Aharoni A , Keizer LCP, Blanco-Portales R et al. Novel insight into vascular, stress, and auxin-dependent and -independent gene expression programs in strawberry, a non-climacteric fruit. Plant Physiol. 2002;129:1019–31. 12114557 10.1104/pp.003558 PMC166497

[76] Feng J , Dai C, Luo H et al. Reporter gene expression reveals precise auxin synthesis sites during fruit and root development in wild strawberry. J Exp Bot. 2019;70:563–74. 30371880 10.1093/jxb/ery384 PMC6322568

[77] Mazzoni-Putman SM , Brumos J, Zhao C et al. Auxin interactions with other hormones in plant development. Cold Spring Harb Perspect Biol. 2021;13. 10.1101/cshperspect.a039990 PMC8485746 33903155

[78] Caruana JC , Sittmann JW, Wang W et al. Suppressor of Runnerless encodes a DELLA protein that controls runner formation for asexual reproduction in strawberry. Mol Plant. 2018;11:230–3. 29158170 10.1016/j.molp.2017.11.001

[79] Sugiura A , Inaba A. Studies on the mechanism of gibberellin-induced seedlessness of Delaware grapes. I. Effect of pre-bloom gibberellin treatment on pollen germination. Engei Gakkai Zasshi. 1966;35:233–41.

[80] Kimura PH , Okamoto G, Hirano K. Effects of gibberellic acid and streptomycin on pollen germination and ovule and seed development in Muscat bailey a. Am J Enol Viticult. 1996;47:152–6.

[81] Agüero C , Vigliocco A, Abdala G et al. Effect of gibberellic acid and uniconazol on embryo abortion in the stenospermocarpic grape cultivars Emperatriz and Perlon. Plant Growth Regul. 2000;30:9–16.

[82] Acheampong AK , Zheng C, Halaly T et al. Abnormal endogenous repression of GA signaling in a seedless table grape cultivar with high berry growth response to GA application. Front Plant Sci. 2017;8:850. 28596775 10.3389/fpls.2017.00850 PMC5442209

[83] He H , Liang G, Lu S et al. Genome-wide identification and expression analysis of GA2ox, GA3ox, and GA20ox are related to gibberellin oxidase genes in grape (*Vitis Vinifera* L.). Genes. 2019;10:680. 10.3390/genes10090680 PMC6771001 31492001

[84] Wang W , Bai Y, Koilkonda P et al. Genome-wide identification and characterization of gibberellin metabolic and signal transduction (GA MST) pathway mediating seed and berry development (SBD) in grape (Vitis vinifera L.). BMC Plant Biol. 2020;20:384. 32825825 10.1186/s12870-020-02591-1 PMC7441673

[85] Weaver RJ , Mccune SB. Further studies with gibberellin on Vitis vinifera grapes. Bot Gaz. 1960;121:155–62.

[86] Lu L , Liang J, Zhu X et al. Auxin- and cytokinin-induced berries set in grapevine partly rely on enhanced gibberellin biosynthesis. Tree Genet Genomes. 2016;12:41.

[87] Parada F , Espinoza C, Arce-Johnson P. InTech. In: El-Esawi M, ed. Phytohormones–Signaling Mechanisms and Crosstalk in Plant Development and Stress Responses. USA: InRech press, 2017.

[88] Gogoy F , Kuhn N, Muñoz M et al. The role of auxin during early berry development in grapevine as revealed by transcript profiling from pollination to fruit set. Hortic Res. 2021;8:140. 34127649 10.1038/s41438-021-00568-1 PMC8203632

[89] Jung CJ , Hur YY, Yu HJ et al. Gibberellin application at pre-bloom in grapevines Down-regulates the expressions of *VvIAA9* and *VvARF7*, negative regulators of fruit set initiation, during Parthenocarpic fruit development. PLoS One. 2014;9:e95634. 24743886 10.1371/journal.pone.0095634 PMC3990702

[90] Wang C , Jogaiah S, Zhang W et al. Spatio-temporal expression of *miRNA159* family members and their GAMYB target gene during the modulation of gibberellin-induced grapevine parthenocarpy. J Exp Bot. 2018;69:3639–50. 29905866 10.1093/jxb/ery172

[91] Weiss D , Ori N. Mechanisms of cross talk between gibberellin and other hormones. Plant Physiol. 2007;144:1240–6. 17616507 10.1104/pp.107.100370 PMC1914132

[92] Bai MY , Shang JX, Oh E et al. Brassinosteroid, gibberellin and phytochrome impinge on a common transcription module in Arabidopsis. Nat Cell Biol. 2012;14:810–7. 22820377 10.1038/ncb2546 PMC3606816

[93] Hou X , Li Y, Xia K et al. DELLAs modulate jasmonate signaling via competitive binding to JAZs. Dev Cell. 2010;19:884–94. 21145503 10.1016/j.devcel.2010.10.024

